# Long Non-coding RNAs and mRNAs Expression Profiles of Monocyte-Derived Dendritic Cells From PBMCs in AR

**DOI:** 10.3389/fcell.2021.636477

**Published:** 2021-02-09

**Authors:** Yumei Zhou, Xuemei Chen, Yanfei Zheng, Rongmin Shen, Shuxian Sun, Fei Yang, Jiayu Min, Lei Bao, Yan Zhang, Xiaoshan Zhao, Ji Wang, Qi Wang

**Affiliations:** ^1^National Institute of TCM Constitution and Preventive Medicine, School of Chinese Medicine, Beijing University of Chinese Medicine, Beijing, China; ^2^School of Traditional Chinese Medicine, Southern Medical University, Guangzhou, China

**Keywords:** monocyte-derived dendritic cells, allergic rhinitis, long non-coding RNA, immunoregulation, mRNA

## Abstract

**Objective:**

The objective of this study is to explore the long non-coding RNAs (lncRNAs) and messenger RNAs (mRNAs) expression profiles of monocyte-derived dendritic cells (DCs) obtained from peripheral blood mononuclear cells (PBMCs). DCs are known to play a major role in the regulating function of allergic rhinitis (AR).

**Methods:**

PBMCs were separately isolated from the human peripheral blood of patients with AR and normal person (NP). The mixed lymphocyte reaction (MLR) assay was used to evaluate the function of DCs. Flow cytometry was used to determine the immune regulatory function of immature DCs (imDCs) and mature DCs (mDCs). lncRNAs and mRNAs in the NP group (DCs isolated from NP) and the test group (DCs isolated from patients with AR) were identified via chip technology and bioinformatic analyses. Moreover, bioinformatic analyses were employed to identify the related biological functions of monocyte-derived DCs and construct the functional networks of lncRNAs and mRNAs that are differentially expressed (DE) in imDCs and mDCs.

**Results:**

MLR was significantly higher in the mDCs group than that in the imDCs group. CD14 was highly expressed in imDCs, whereas HLA-DR, CD80, and CD86 were highly expressed in mDCs (*p* < 0.001). We identified 962 DE lncRNAs and 308 DE mRNAs in the imDCs of NP and patients with AR. Additionally, there were 601 DE lncRNAs and 168 DE mRNAs in the mDCs in the NP and test groups. Quantitative RT-qPCR was used to study the significant fold changes of lncRNAs and mRNAs. The Kyoto Encyclopedia of Genes and Genomes (KEGG) analysis found 16 significant regulated pathways in imDCs and 10 significant regulated pathways in mDCs, including the phagosome, cell adhesion signaling pathway, and inflammatory mediator regulation of TRP channels pathway.

**Conclusion:**

Our research studied the lncRNA and mRNA expression profiles of monocyte-derived DCs and demonstrated the functional networks that are involved in monocyte-derived DCs-mediated regulation in AR. These results provided possible molecular mechanisms of monocyte-derived DCs in the immunoregulating function and laid the foundation for the molecular therapeutic targets of AR.

## Background

Allergic rhinitis (AR) is a very common allergic disease that affects 10–40% of the global population (Bousquet et al., 2008). Its remarkable prevalence and relapses put an extensive burden on its patients and the society. Furthermore, AR negatively impacts the quality of life of patients with AR. There had been a marked increase in the prevalence of AR during the past years (Wang et al., 2016). AR turns into asthma if it is not treated in time, and the adequate treatment of AR can alleviate the severity of asthma (Leynaert et al., 2004). Researchers have illustrated that mast cell infiltration, lymphocytes imbalance, and goblet cell hyperplasia are involved in the pathogenesis of AR (Ouyang et al., 2010; Poggi et al., 2012). AR, which is a type I allergic disorder that is mediated by IgE humoral immune response, is accompanied by an influx of eosinophils and T helper 2 cells that secrete pro-inflammatory cytokines, namely, IL-4, IL-5, and IL-13 (Wilson et al., 2005). Abnormal innate and adaptive immune responses play a major role in the pathogenesis of AR.

Dendritic cells (DCs), which are the most important antigen-presenting cells (APCs) that send signals to the T cells, mainly participate in the pathogenesis of many diseases with immunoregulatory mechanisms, such as AR. DCs link the innate and adaptive immune responses. The peripheral blood mononuclear cells (PBMCs) have a round nucleus (Delves, 2016). PBMCs include lymphocytes, monocytes, and DCs. In humans, the frequencies of these DCs vary among individuals. PBMCs are divided into various functional subtypes with respect to the specific cytokine expression profiles, surface markers, and the transcription factors. Phenotypic and functional assessments of PBMC research lay the foundation of the human immune system research; hence, the knowledge that population is represented in the peripheral blood and how they act with other immune cells is essential. Additionally, the results from human PBMC studies (Schiekofer et al., 2003; Tacconi et al., 2004; Chang et al., 2014) cannot be neglected. Therefore, it is important to know the progression of AR along with its expression profiles in PBMCs, especially DCs.

Long non-coding RNAs (lncRNAs), over 200 nt in length, is a type of RNA that does not a protein coding function (Ulitsky and Bartel, 2013). These RNAs have been regarded as indispensable epigenetic regulators and are probably involved in the cell’s biological behaviors (Kopp and Mendell, 2018). For example, they are involved in regulating the homeostasis of the immune system (Wang et al., 2014; Du et al., 2017). However, it is critical to find out whether lncRNA can immunoregulate DC in the progression of AR.

The combination of lncRNA–messenger RNA (mRNA) expression profiles and functional networks is adopted to analyze the DC-mediated regulation functions. These results improve our understanding of lncRNAs in the immunoregulatory function of monocyte-derived DCs and indicate the potential targets for the curative treatment of AR.

## Materials and Methods

### Subjects

Patients with AR visited doctors in the outpatient service in the Guo Yi Tang of Beijing University of Chinese Medicine. In this study, there were 24 subjects: 12 males and 12 females. They were divided into two groups: the AR group (patients with AR, 12 subjects: five males and seven females) and NP group (normal persons, 12 subjects: four males and eight females). With support/approval from the Ethics Committee of Beijing University of Chinese Medicine, this study was conducted while adhering to the principles of the Declaration of Helsinki. Patients in the AR group were positive for skin puncture test, including pollen, food, dust mites, paint, or molds as well as in specific IgE. Two weeks before study recruitment, these patients with AR received no topical or systemic corticosteroid therapy. We chose the study participants with no history of smoking or other immune system disorders, such as rheumatoid arthritis, systemic lupus erythematosus, and scleroderma.

### Isolation of PBMCs and Generation of DCs

The whole blood samples obtained from the two groups were stored in vacuum tubes with heparin, and PBMCs were isolated from these samples by lymphocyte separation solution (Tianjin Haoyang Biological Manufacture Co., Ltd.). Mononuclear cells were seeded in 12-well plates with the RPMI 1640 medium that contains 10% heat-inactivated fetal calf serum (FCS, GIBCO, Germany) and 2 mM of L-glutamine (R10 medium, Sigma, St. Louis, MO, United States). After incubation at 37°C for 2 h, the non-adherent cells were removed and the adherent cells were cultured within the medium containing 100 ng/ml of rhGM-CSF and 100 ng/ml of rhIL-4 (R&D Systems, Minneapolis, MN, United States). On the sixth day, 50 ng/ml of TNF-α (R&D Systems, Minneapolis, MN, United States) was added into the samples; the method had the same protocol in the study of Andreia et al. (2005). Immature DCs (imDCs) were collected on the fifth day, and the mature DCs (mDCs) were collected on the seventh day.

### Mixed Lymphocyte Reaction (MLR)

After being treated with 25 μg/mL of mitomycin at 37°C for 30 min, the DCs were placed at the concentrations of 2 × 10^8^ cells per well at a quantity of 200 μl and incubated with non-adherent PBMCs obtained from the same healthy people. These samples were later stimulated by lymphocytes in the concentration proportions of 1:10, 1:50, and 1:100. Thereafter, the samples were mixed and incubated with non-adherent PBMC from the same healthy persons at the same concentrations in triplicate. The cells were treated with 10% fetal bovine serum. Then, 10 μl of 3-(4,5-dimethylthiazol-2-yl)-2,5-diphenyl-2H-tetrazolium bromide (MTT) solution (5 mg/ml, medium dilution, Sigma–Aldrich Chemical Co., St. Louis, MO, United States) was added to each well, and the cells were incubated for 72 h in the incubator. Then, 150 μl of DMSO was added followed by the addition of enzymes after 4 h. The absorbance was detected using a spectrophotometer at 570 nm.

### DC Surface Marker Expression Analysis in imDCs and mDCs

The CD14 (PerCP-Cy 5.5, BD Biosciences, United States), HLA-DR (APC, BD Biosciences, United States), and isotype mouse IgG2a-PE (PE, BioLegend, United States) were added in the samples of imDCs. Moreover, CD86-APC, CD80-PE, and isotype mouse IgG1–FITC (BioLegend, United States) were added in the samples of mDCs. The cells were then suspended with precooled PBS, counted under a microscope, and centrifuged at 1000 *g* for 5 min. Data were acquired using a FACSCalibur cytometer (BD Biosciences, United States) and the ratios of CD14^+^, HLA-DR^+^, CD80^+^, and CD86^+^ DCs were determined.

### RNA Extraction, Labeling, Chip Hybridization, and Scanning

The RNA extraction, labeling, chip hybridization, and scanning were all finished following the use of Agilent Human lncRNA–mRNA profiling chip (4^∗^180K, Design ID: 062918). All the chip results were detailed according to the processes of software operations. The screening criteria were to increase or decrease the fold change value ≥ 2.0 and *p*-value < 0.05.

### Gene Ontology (GO) and Pathway Enrichment Analysis

The GO and Kyoto Encyclopedia of Genes and Genomes (KEGG) enrichment analysis were used to study the differentially expressed (DE) mRNAs. The results of the target gene analysis of lncRNA and the mRNA expression results on the chip need to be correlated so that the upregulated and downregulated DE genes can be investigated. The corrected *p*-value < 0.05 by calculating the FDR and FDR < 0.05 was selected as the threshold.

### LncRNA–mRNA-Weighted Co-expression Network

The correlation of the lncRNA–mRNA expression in the imDCs and mDCs was calculated. Then, the relationship pairs of LncRNA and mRNA based on the abovementioned criteria (*p*-value < 0.05, FDR < 0.05) were screened. The co-expression network of lncRNAs and mRNAs was constructed, and the co-expression network of lncRNAs and mRNAs was then established.

### Real-Time PCR Verification of DE Genes

Quantitative RT-qPCR was used to investigate the different expressions of imDCs’ and mDCs’ genes between the patients with AR and NP. The total RNA was extracted from imDCs and DCs according to the kit for cells. In all, 20 μg of total RNA was converted into cDNA by using oligo (dT) and reverse transcriptase (Thermo, United States) to analyze the qPCR results. The thermal cycler conditions were set as follows: amplificated at 95°C for 10 min, 95°C for 15 s, 60°C for 60 s, 40 cycles of denaturation (15 s, 94°C), 15 s at 95°C, and a combined process of annealing and extension (1 min, 60°C). Supplementary Table S1 shows the primers for these genes.

### Statistical Analysis

The SPSS 22.0 software was used for statistical analysis in this study. Results were expressed as mean ± standard deviation. The relationship of lncRNAs and mRNAs was determined by the Spearman’s correlation coefficient. *p*-values < 0.05 were considered significant values for this study.

## Results

### Allogeneic T-Cell Proliferation Experiment

**FIGURE 1:**
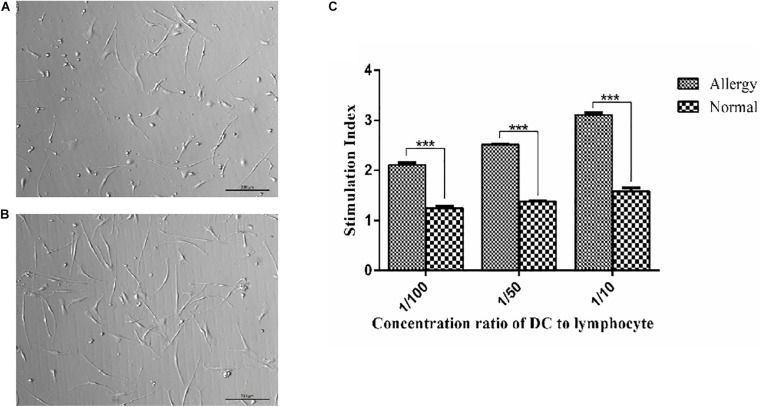
Morphology of dendritic cells and the functional activity of DCs were assayed by autologous MLR. **(A,B)** imDCs and mDCs, mDCs have a more structure of dendrites. **(C)** MLR was determined by the stimulation index. 2 × 10^8^ ml^–1^ DCs and lymphocytes were cultured with the medium for 72 h, and then DCs were collected and autologous MLR was performed. The control group was stimulated with 25 mg/L mitomycin as controls. ***P < 0.001.

In allergenic mixed lymphocyte reaction (MLR), the levels of T-cell proliferation were increased with the proportion of T cells. DCs in the patients with AR have a stronger stimulation ability than NP as shown in Figures 1A–C. When the ratio of DC cells to T cells was 1:50 and 1:100, the difference was significant (*p* < 0.001).

### Immunophenotype of DCs in Patients With AR and NP

**FIGURE 2:**
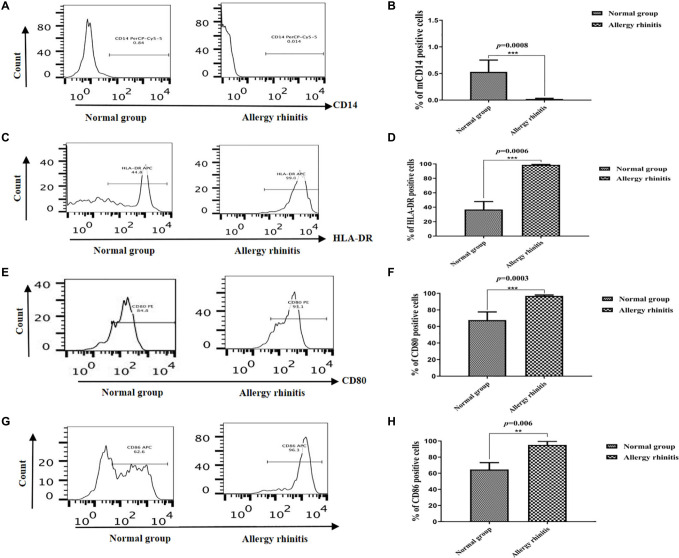
Flow cytometric analysis showing the percentage of CD14, HLA-DR, CD80, and CD86 in DCs. **(A,B)** The flow cytometric analysis showed that percentage of CD14 in NP group was lower than that in AR group (*p* = 0.0008). **(C,D)** The flow cytometric analysis showed that percentage of HLA-DR in NP group was lower than that in AR group. **(E,F)** The flow cytometric analysis showed that percentage of CD80 in NP group was lower than that in AR group. **(G,H)** The flow cytometric analysis showed that percentage of CD86 in NP group was lower than that in AR group. ***P < 0.001, **P < 0.01.

We evaluated the percentages of CD14, HLA-DR, CD80, and CD86 in the DCs. The immunophenotypic characteristics of DCs in patients with AR were compared with that of NP, as shown in Figure 2. The CD14 concentration of imDCs was lower in patients with AR than that in the NP group. The mean percentage of CD14^+^ DCs in the patients with AR was 0.84 ± 0.25% (median: 0.78%), and it was significantly lower than the value in the NP group (*p* = 0.0008), where the mean percentage of these cells was 0.012 ± 0.013% (median: 0.017%) (Figures 2A,B).

The mean proportion of HLA-DR^+^ mDCs in the patients with AR was 44.25 ± 8.64% (median: 44.8%) and was higher (*p* = 0.0006) than the NP group, where the mean percentage of these cells was 95.3 ± 3.84% (median: 99.0%) (Figures 2C,D).

The mean proportion of CD80^+^ mDCs in the patients with AR was 72.15 ± 7.64% (median: 67.78%) and was higher (*p* = 0.0003) than the NP group, where the mean percentage of these cells was 95.3 ± 3.84% (median: 96.92%) (Figures 2E,F).

The mean proportion of CD86^+^ mDCs in the patients with AR was 62.15 ± 7.64% (median: 64.69%) and was higher (*p* = 0.006) than the control group, where the mean percentage of these cells was 92.3 ± 5.84% (median: 95.12%) (Figures 2G,H).

### Identification of DE lncRNAs and mRNAs in imDCs

**FIGURE 3:**
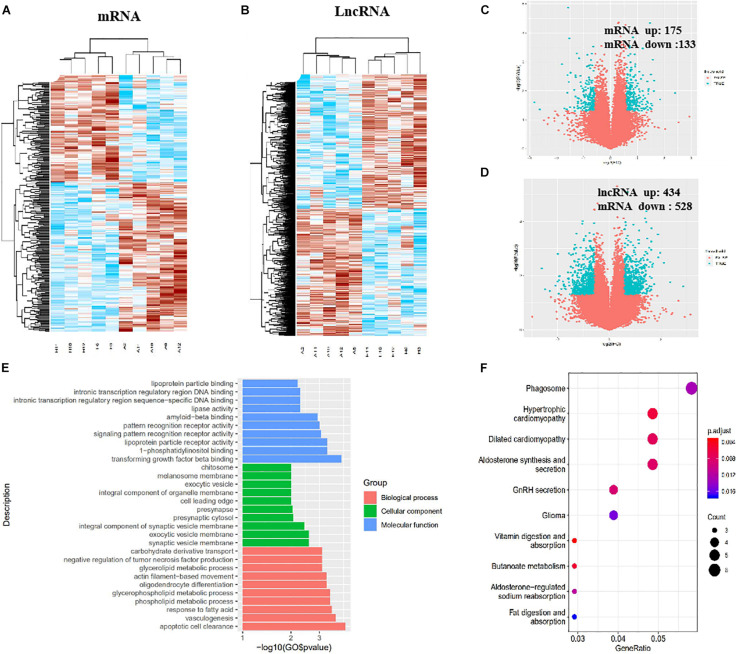
Identification of DE lncRNAs and mRNAs, GO, and KEGG analysis in imDCs. **(A)** Heatmaps of DE mRNAs between the AR group and NP group. **(B)** Heatmaps of DE lncRNAs between the AR group and NP group. **(C)** Volcano plots of DE mRNAs between the AR group and NP group. **(D)** Volcano plots of DE lncRNAs between the AR group and NP group. **(E)** Top 10 terms from a GO analysis of molecular function, biological process, and cellular component. **(F)** DE mRNAs were clustered by KEGG analysis, and the top 10 pathways are shown.

**FIGURE 4:**
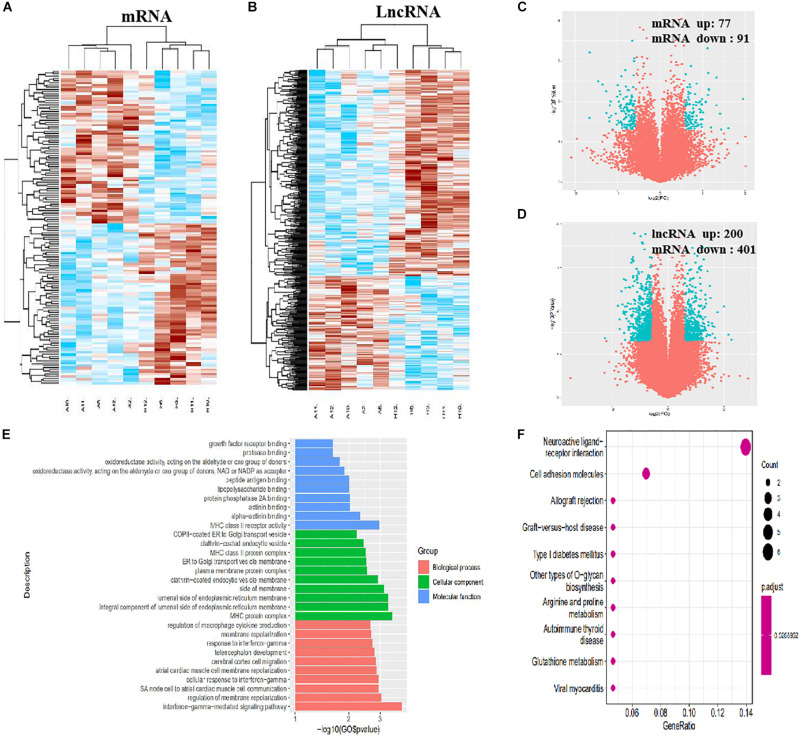
Identification of DE lncRNAs and mRNAs, GO, and KEGG analysis in mDCs. **(A)** Heatmaps of DE mRNAs between the AR group and NP group. **(B)** Heatmaps of DE lncRNAs between the AR group and NP group. **(C)** Volcano plots of DE mRNAs between the AR group and NP group. **(D)** Volcano plots of DE lncRNAs between the AR group and NP group. **(E)** Top 10 terms from a GO analysis of molecular function, biological process, and cellular component. **(F)** DE mRNAs were clustered by KEGG analysis, and the top 10 pathways are shown.

In total, 308 DE mRNAs, including 175 upregulated mRNAs and 133 downregulated mRNAs, were found in the imDCs of patients with AR and NP. A clustergram (Figure 3A) and volcano plots (Figure 3C) are used to depict DE mRNAs. Table 1 shows 67 mRNAs with the largest fold changes. The list contains several genes, including HLA-C, MARCO, KIR2DS3, ITGAV, CD36, and IFNB1. Additionally, 168 DE mRNAs, including 77 upregulated mRNAs and 91 downregulated mRNAs, were found in the mDCs of patients with AR. Figures 4A,C show the clustergram and volcano plots of the DE mRNAs, respectively. Table 2 shows the 10 mRNAs with the greatest variation, and several genes, such as HLA-B, F11R, HLA-DQB1b, HLA-DQB1, and PTAFR, are also shown.

**TABLE 1:** The characteristics of mRNAs with the largest fold change in imDCs.

GeneName	Genbank Accession	FC (abs)	Regulation
Fatty acid 2-hydroxylase	NM_024306	2.7454393	Up
Uncharacterized LOC645984	AK095436	2.289749	Up
Shisa homolog 9 (Xenopus laevis)	NM_001145205	2.3817623	Down
NK2 homeobox 1	NM_003317	2.2473493	Down
Sodium channel, voltage-gated, type VII, alpha	NM_002976	3.4931335	Down
Nucleolar and spindle associated protein 1	NM_016359	2.0980568	Up
Ventral anterior homeobox 1	NM_199131	2.798632	Down
Chromosome 20 open reading frame 132	NM_213631	2.831788	Up
Programmed cell death 1 ligand 2	NM_025239	3.0216887	Down
Solute carrier family 29 (nucleoside transporters), member 1	NM_001078177	2.2991307	Up
Very low density lipoprotein receptor	NM_001018056	2.4687624	Down
Tropomyosin 2 (beta)	NM_213674	2.175945	Down
Uncharacterized LOC100131129	AK127184	2.1942973	Up
Peptidyl arginine deiminase, type II	NM_007365	3.0724247	Up
CD300 molecule-like family member f	NM_139018	2.5870113	Up
Programmed cell death 1 ligand 2	NM_025239	2.9215207	Down
CD36 molecule (thrombospondin receptor)	NM_001001547	2.167322	Down
Suppression of tumorigenicity 5	NM_005418	2.673464	Up
T-cell acute lymphocytic leukemia 1	NM_003189	2.3056405	Down
Fc receptor-like B	NM_001002901	2.3118186	Up

**TABLE 2:** The top 10 co-expression of mRNA and lncRNA in imDCs.

mRNA	Gene	lncRNA	Gene	Correlation coefficient	*P*-value
NM_018208	ETNK2	NON-HSAT008926	ETNK2	0.9970621	3.24775E–10
NM_005891	ACAT2	NON-HSAG045301	NON-HSAG045301	0.9953173	2.09182E–09
NM_182985	TRIM69	NR_104175.1	LOC400799	−0.9914054	2.36259E–08
BX538082	GPR17	FR351114	FR351114	0.9906462	3.31169E–08
NM_002977	SCN9A	NON-HSAG029733	NON-HSAG029733	0.9882097	8.33527E–08
NM_014485	HPGDS	NON-HSAT097445	HPGDS	0.9877657	9.6583E–08
AK131565	LOC100132368	ENST00000588609	LINC00906-004	0.9863838	1.47941E–07
NM_005005	NDUFB9	NON-HSAT101913	RP11-1113N2.4	0.9860654	1.62207E–07
XM_001719518	LOC100128869	NON-HSAG042749	NON-HSAG042749	−0.985726	1.78529E–07
NM_032772	ZNF503	ENST00000438293	RP11-88H9.2-003	0.9853318	1.98986E–07

In total, 962 DE lncRNAs, including 434 upregulated and 528 downregulated lncRNAs, were found in patients with AR and NP. The clustergram (Figure 3B) and volcano plots (Figure 3D) show DE lncRNAs. In total, 601 lncRNAs, including 200 upregulated and 401 downregulated lncRNAs, were found in the DE mDCs of the patients with AR. The clustergram in Figure 4B and the volcano plots in Figure 4D show the DE lncRNAs. Tables 1, 3 show the top 10 lncRNAs of imDCs and mDCs with the largest fold changes. The pathways, including interferon-gamma-mediated signaling pathway, membrane repolarization, and peptide antigen binding, that contribute to the phagocytosis function in imDCs and antigen-presenting function of mDCs were also identified.

**TABLE 3:** The characteristics of mRNAs with the largest fold change in mDCs.

GeneName	Accession no.	Fold change	Regulation
Armadillo repeat containing 9	NM_025139	2.1053998	Up
Cat eye syndrome chromosome region, candidate 2	NM_031413	2.1667209	Down
Semenogelin II	NM_003008	2.2934558	Up
Polycystic kidney disease 1 like 1	NM_138295	3.8540776	Up
Rho guanine nucleotide exchange factor (GEF) 35	NM_001003702	2.662969	Up
Sushi domain containing 4	NM_017982	3.1600573	Down
ST6 beta-galactosamide alpha-2,6-sialyltranferase 1	NM_173216	2.504244	UP
Chromosome 2 open reading frame 71	NM_001029883	2.1819167	Up
Dehydrogenase/reductase (SDR family) member 3	NM_004753	2.2156718	Up
Sterile alpha motif and leucine zipper containing kinase AZK	NM_016653	2.2057335	UP
Chromosome 5 open reading frame 62	NM_032947	2.0991595	Up
Transmembrane protein ENSP00000343375	BC031304	2.0236611	Down
Family with sequence similarity 101, member B	NM_182705	2.506473	Up
Olfactory receptor, family 2, subfamily T, member 8	NM_001005522	2.5608928	Down
Sorbin and SH3 domain containing 1	NM_001034954	2.0617623	Down
Transmembrane protease, serine 6	BC039082	2.2889757	Down
DEAD (Asp-Glu-Ala-Asp) box polypeptide 6	NM_004397	2.0167336	Up
spondin 2, extracellular matrix protein	NM_012445	2.7114112	Up
HLA-DBQ1	NM_001243961	2.154	Down

### Interaction, Co-expression Network Analysis of DE mRNAs in Patients With AR

**FIGURE 5:**
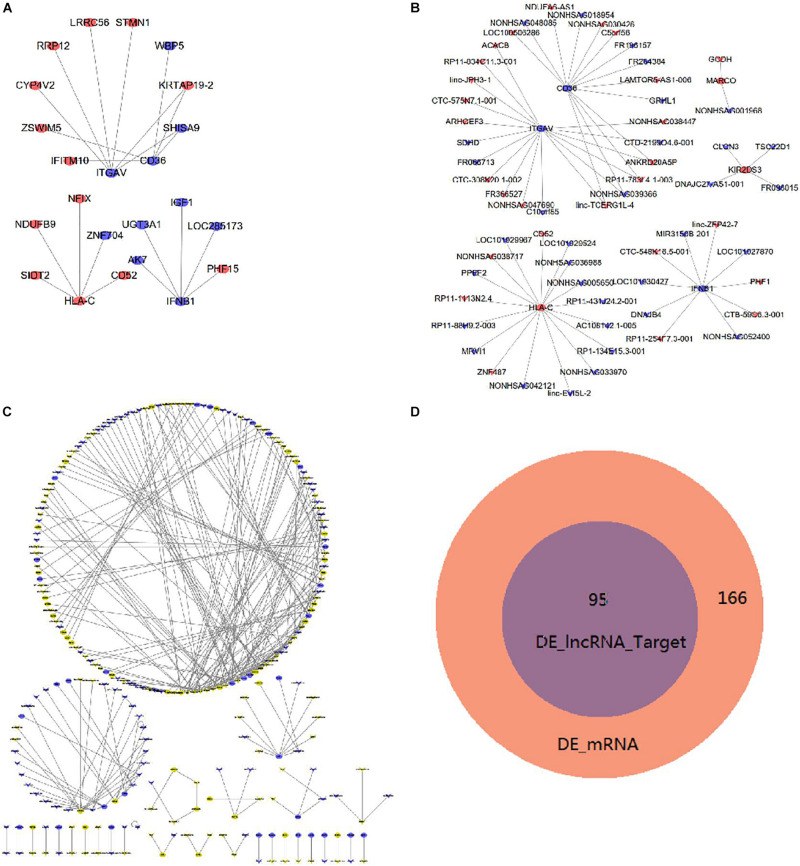
Interaction and co-expression network analysis, analysis of lncRNA target mRNAs in imDCs. **(A)** Interactions between DE mRNAs. Purple indicates upregulated genes, and green indicates downregulated genes. **(B)** Co-expression network of DE lncRNAs and DE mRNAs. **(C)** DE lncRNAs and their target genes with a combined score larger than 0.9 are shown. Yellow indicates upregulated genes, and blue indicates downregulated genes. **(D)** Venn diagram showing DE mRNAs and target genes of DE lncRNAs.

Figure 5A shows the interactions of proteins that were coded by DE mRNAs in imDCs. Additionally, Figure 5B shows the co-expression network between DE lncRNAs and mRNAs. TRIM69 has the maximum target mRNAs including 58 DE mRNAs, and SIDT2 has the maximum co-expressed lncRNAs. Table 2 shows the top 10 co-expression pairs in imDCs. lncRNAs exert their biological function as ceRNAs (Wilfried et al., 2016).

We identified 268 target genes after analyzing the possible DE lncRNAs target genes in imDCs. Figure 5C shows the target genes with a combined score of more than 0.9. HLA-C was the target gene of AC108142.1-005, and CD36 was the target gene of FR264384. Moreover, IFNB1 was the target gene of MIR3150B-210. The Venn diagram analysis showed that 95 mRNAs were coincided between the 166 DE mRNAs and the 95 DE lncRNA target genes (Figure 5D). The 95 DE lncRNAs were all included in the 166 DE mRNAs.

**FIGURE 6:**
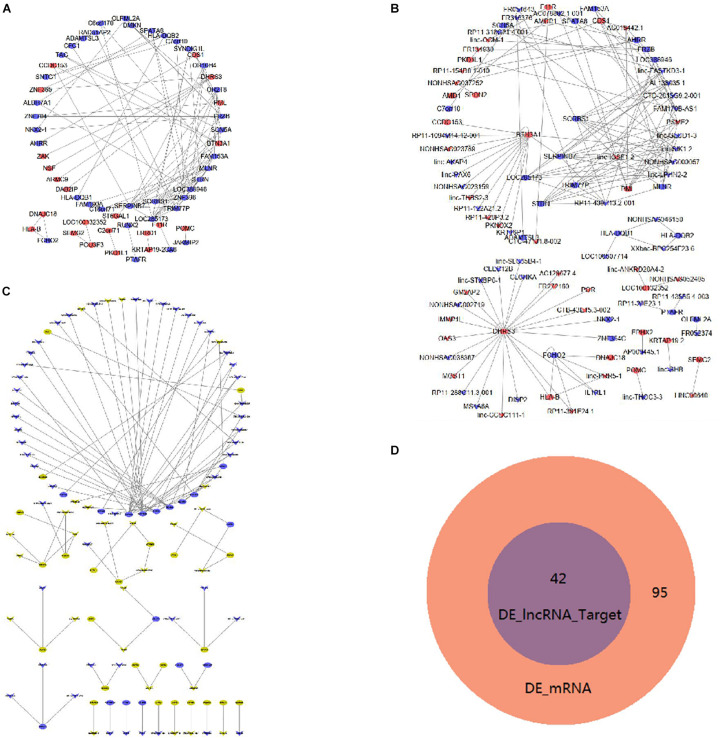
Interaction and co-expression network analysis, analysis of lncRNA target mRNAsin mDCs. **(A)** Interactions between DE mRNAs. Purple indicates upregulated genes, and green indicates downregulated genes. **(B)** Co-expression network of DE lncRNAs and DE mRNAs. **(C)** DE lncRNAs and their target genes with a combined score larger than 0.9 are shown. Yellow indicates upregulated genes, and blue indicates downregulated genes. **(D)** Venn diagram showing DE mRNAs and target genes of DE lncRNAs.

Figure 6A shows the interaction proteins that were coded by DE mRNAs in mDCs. In this network, HLA-B, HLA-DQB1, HLA-DQB2, PTAFR, and F11R are important genes that interact with many other DE mRNAs. Furthermore, Figure 6B shows the co-expression network of DE lncRNAs and mRNAs. TRIM77P has the maximum target numbers including 39 DE mRNAs, in which FAM153A and ZNF396 have the maximum co-expressed lncRNAs. Table 4 shows the top 10 co-expression pairs of mDCs.

**TABLE 4:** The top 10 co-expression of mRNA and lncRNA in mDCs.

mRNA	Gene	lncRNA	Gene	Correlation coefficient	*P*-value
NM_012445	SPON2	NON-HSAG037252	NON-HSAG037252	0.9947499	3.30E–09
NM_138782	FCHO2	NON-HSAT102095	FCHO2	0.9945368	3.87E–09
AK311167	LOC100132352	TCONS_l2_00028804	linc-ANKRD20A4-2	0.9832951	3.34E–07
NM_003162	STRN	ENST00000562064	CTD-2015G9.2-001	0.9825929	3.93E–07
NM_001034954	SORBS1	TCONS_00010265	linc-FASTKD3-1	0.9794563	7.60E–07
NM_001463	FRZB	NR_038973.1	FAM170B-AS1	0.9777894	1.04E–06
NM_001979	EPHX2	NON-HSAT021480	AP000445.1	−0.9769811	1.19E–06
AB018295	FAM153A	TCONS_00002078	linc-LPHN2-2	0.9747958	1.71E–06
NM_007048	BTN3A1	NON-HSAT142393	RP11-429P3.2	0.9737392	2.02E–06
NM_182487	OLFML2A	FR052374	FR052374	0.9723832	2.46E–06
NM_001146162	TRIM77P	ENST00000541885	RP11-439H13.2-001	0.9723254	2.48E–06

Additionally, we also investigated the possible presence of DE lncRNA target genes in mDCs. In our study, 99 target genes were identified in these DE lncRNAs. Figure 6C shows the target genes with the combined score of more than 0.9. In the figure, HLA-B has three target genes of lincRNAs, namely, DHRS3, FCHO2, and linc-PRR5-1. Figure 6D shows that 42 mRNAs coincided between the 95 DE mRNAs and the 42 DE lncRNA target genes in the Venn diagram analysis. Moreover, the 42 DE lncRNAs were included in the 95 DE mRNAs. It also includes some known inflammatory-related molecules.

### Validation of DE mRNA and lncRNA Expression Levels by RT-qPCR

**FIGURE 7:**
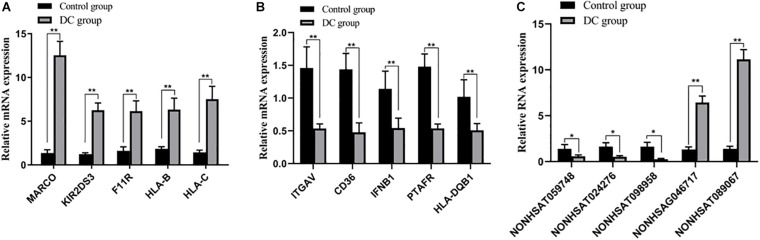
Validation of DE mRNAs and lncRNAs. **(A,B)** DE mRNAs were confirmed by qPCR. **(C)** DE lncRNAs were confirmed by qPCR. Ligand 1, MACO, the scavenger receptor; Ligand 2, KIR2DS3, a type of killer cell immunoglobulin-like receptors (KIRs); Ligand 3, F11R, a cell adhesion molecule found on the surface of human platelets; Ligand 4 and Ligand 5, HLA-B and HLA-C are all a type of human Leukocyte Antigen (HLA) class I molecules; Ligand 6, ITGAV, integrin subunit alpha v; Ligand 7, CD36, a membrane glycoprotein present on platelets, mononuclear phagocytes, adipocytes, hepatocytes, myocytes, and some epithelia; Ligand 8, IFNB1, interferon beta, a secreted cytokine; Ligand 9, PTAFR, platelet-activating factor receptor; Ligand 10, HLA-DQB1, human leukocyte antigen DQB1. **P < 0.01, *P < 0.05.

RT-qPCR was used to evaluate DE mRNAs and lncRNAs to verify our RNA chip results. We randomly detected three lncRNAs and 10 mRNAs. MARCO, KIR2DS3, F11R, HLA-B, HLA-C, NON-HSAG046717, and NON-HSAT089067 were upregulated, whereas ITGAV, CD36, IFNB1, PTAFR, HLA-DQB1, NON-HSAT 059748, NON-HSAT024276, and NON-HSAT098958 were downregulated. The results of RT-qPCR were consistent with those of RNA chip results, hence confirming that our chip data were reliable (Figure 7).

## Discussion

In our research, we used rhGM-CSF, rhIL-4, and TNF-α to induce the mDCs from PBMCs. The generation of human monocyte-derived DCs from whole blood was recognized by all the scientists (Wilfried et al., 2016). We investigated phenotypic and functional features of DE DCs *in vitro* from patients with AR and the NPs. Besides that, we used RT-qPCR to confirm these findings. Our KEGG pathway analysis (Tables 5, 6) indicates that interferon-gamma-mediated signaling pathway, membrane repolarization, and peptide antigen binding pathways contribute to the phagocytosis function in imDCs, and the antigen-presenting function in mDCs contributes to the immunoregulatory function of DCs in AR.

**TABLE 5:** Pathways KEGG analysis of imDCs.

Pathway	Count	*P*-value	Corrected *P*-value	Gene
Vitamin digestion and absorption	3	0.003377492	0.355867536	PLB1/SLC19A1/SCARB1
Butanoate metabolism	3	0.005268382	0.355867536	AACS/ACADS/ACAT2
Hypertrophic cardiomyopathy	5	0.005764726	0.355867536	TPM2/TPM1/MYH7/ITGAV/IGF1
Dilated cardiomyopathy	5	0.00755082	0.355867536	TPM2/TPM1/MYH7/ITGAV/IGF1
Aldosterone synthesis and secretion	5	0.008223347	0.355867536	CACNA1I/KCNJ5/PRKCB/SCARB1/POMC
GnRH secretion	4	0.008896688	0.355867536	CACNA1I/KCNJ5/PRKCB/PIK3R3
Aldosterone-regulated sodium reabsorption	3	0.011502085	0.390782867	PRKCB/PIK3R3/IGF1
Phagosome	6	0.013026096	0.390782867	C1R/ITGAV/SCARB1/MARCO/CD36/HLA-C
Glioma	4	0.015303573	0.408095288	PRKCB/PIK3R3/CDK6/IGF1
Fat digestion and absorption	3	0.017291278	0.414990664	SCARB1/CD36/ACAT2
Valine, leucine, and isoleucine degradation	3	0.023152058	0.479306938	AACS/ACADS/ACAT2
Cholesterol metabolism	3	0.025762258	0.479306938	LRPAP1/SCARB1/CD36
Natural killer cell-mediated cytotoxicity	5	0.025962459	0.479306938	IFNB1/PRKCB/PIK3R3/KIR2DS3/HLA-C
Terpenoid backbone biosynthesis	2	0.031653591	0.542632982	MVD/ACAT2
Inflammatory mediator regulation of TRP channels	4	0.038848883	0.574014469	PRKCB/PIK3R3/IGF1/P2RY2
Parathyroid hormone synthesis, secretion, and action	4	0.046482901	0.574014469	MMP14/RUNX2/PRKCB/PDE4D

**TABLE 6:** Pathways of mDCs with the largest significant difference in KEGG analysis.

Pathway	Count	*P*-value	Corrected *P*-value	Gene
Neuroactive ligand–receptor interaction	6	0.008916046	0.526895235	PTAFR, POMC, OPRM1, MCHR2, GNRH1, MLNR
Allograft rejection	2	0.017316097	0.526895235	HLA-DQB1, HLA-B
Graft-versus-host disease	2	0.020925187	0.526895235	HLA-DQB1, HLA-B
Type I diabetes mellitus	2	0.021872484	0.526895235	HLA-DQB1, HLA-B
Other types of O-glycan biosynthesis	2	0.025835563	0.526895235	ST6GAL1, LFNG
Arginine and proline metabolism	2	0.028984793	0.526895235	AMD1, ALDH7A1
Autoimmune thyroid disease	2	0.032279732	0.526895235	HLA-DQB1, HLA-B
Glutathione metabolism	2	0.036890658	0.526895235	GSTM3, G6PD
Viral myocarditis	2	0.04050554	0.526895235	HLA-DQB1, HLA-B
Cell adhesion molecules	3	0.044652139	0.526895235	HLA-DQB1, HLA-B, F11R
Retinol metabolism	2	0.050761992	0.544537736	DHRS3, ALDH1A1

Dendritic cells play a central role in allergic inflammation (Froidure et al., 2016). The latest *in vitro* techniques allow the *in vitro* differentiation of DCs (Thurner et al., 1999). Their ability to induce the proliferation of T cells in the MLR assay is commonly used for evaluating their functions (Cao et al., 2004). In MLR experiment, the stimulation index of mDCs was significantly higher than that of the imDCs. In our study, the expression of HLA-DR, CD80, and CD86 in patients with AR is indeed upregulated than that in NP, which is a sign of improved mDCs antigen presentation in patients with AR. This result is in accordance with a previous study by KleinJan et al. (2006). In the study, CD14 in the NP group was higher than that in patients with AR. CD14 is the marker of monocytes whose expression decreases gradually during DC differentiation from monocytes. In fact, CD80 and CD86 are important co-stimulating factors that affect the proliferation of T lymphocytes in the DCs (Duperrier et al., 2000; Ebner et al., 2001; Andreia et al., 2005; Wilfried et al., 2016). DCs are the most efficient APCs. It can present the antigens to the T cells for stimulating the adaptive immune response.

More number of studies have focused on exploring the mRNA expressed in DCs, but none have been conducted to reveal which and how mRNA affects DCs in patients with AR. To investigate the mechanism of DCs’ functions in patients with AR, the mRNA expression profile was determined and bioinformatics analysis was performed in our study. In total, 308 mRNAs were identified in the analysis. Among these DE genes, HLA-C, ITGAV, MARCO, CD36, IFNB1, and KIR2DS3 were found to be most significantly upregulated in the imDCs’ network. HLA-C plays an important role in promoting differential DC maturation (Raj et al., 2011). ITGAV is the expression of DC-specific transmembrane protein. MARCO promotes TLR activation, which validates a major role of MARCO in mounting an inflammatory response (Haydn et al., 2014). ImDCs play an important role in the phagocytosis of apoptotic cells, in particular, CD36 (Albert et al., 1998). Additionally, the GO analysis demonstrated specific molecular functions, for example, binding of transforming growth factor-beta, signaling pattern recognition receptor activity, and pattern recognition receptor activity, thereby indicating the critical role of these cytokines in the immunoregulatory functions of imDCs. The KEGG analysis identified 17 signaling pathways of the DE mRNAs, wherein interferon-gamma-mediated signaling pathway, membrane repolarization, and peptide antigen binding were the pathways with the significant differences that contributed to the phagocytosis function of imDCs (Figures 3E,F). This result is consistent with that of a previous study (Lambrecht, 2001). Although research works have been conducted to determine the functions of imDCs in AR, the role of IFNB1 and KIR2DS3 in imDCs was not known in AR (Tables 7, 8).

**TABLE 7:** GO analysis of DE mRNA in imDCs.

Term	Domain	Count	*P*-value	Corrected *P*-value
Apoptotic cell clearance	Biological process	5	4.46014E–05	4.46014E–05
Vasculogenesis	Biological process	6	0.000164507	0.000164507
Response to fatty acid	Biological process	6	0.000263738	0.000263738
Phospholipid metabolic process	Biological process	13	0.000319148	0.000319148
Glycerophospholipid metabolic process	Biological process	11	0.000323952	0.000323952
Oligodendrocyte differentiation	Biological process	6	0.000454451	0.000454451
Actin filament-based movement	Biological process	7	0.00047494	0.00047494
Glycerolipid metabolic process	Biological process	12	0.000748476	0.000748476
Negative regulation of tumor necrosis factor production	Biological process	5	0.000769209	0.000769209
Carbohydrate derivative transport	Biological process	5	0.000769209	0.000769209
Synaptic vesicle membrane	Cellular component	5	0.002649207	0.002649207
Exocytic vesicle membrane	Cellular component	5	0.002649207	0.002649207
Integral component of synaptic vesicle membrane	Cellular component	3	0.003805305	0.003805305
Presynaptic cytosol	Cellular component	2	0.008553871	0.008553871
Presynapse	Cellular component	11	0.008923316	0.008923316
Cell leading edge	Cellular component	10	0.009798418	0.009798418
Integral component of organelle membrane	Cellular component	6	0.009895744	0.009895744
Exocytic vesicle	Cellular component	6	0.009895744	0.009895744
Melanosome membrane	Cellular component	2	0.00990825	0.00990825
Chitosome	Cellular component	2	0.00990825	0.00990825
Transforming growth factor beta binding	Molecular function	4	7.69207E–05	7.69207E–05
1-Phosphatidylinositol binding	Molecular function	3	0.000452198	0.000452198
Lipoprotein particle receptor activity	Molecular function	3	0.000452198	0.000452198
Signaling pattern recognition receptor activity	Molecular function	3	0.000824225	0.000824225
Pattern recognition receptor activity	Molecular function	3	0.000980996	0.000980996
Amyloid-beta binding	Molecular function	5	0.001167764	0.001167764
Lipase activity	Molecular function	5	0.005214971	0.005214971
Intronic transcription regulatory region sequence-specific DNA binding	Molecular function	2	0.005236034	0.005236034
Intronic transcription regulatory region DNA binding	Molecular function	2	0.005236034	0.005236034
Lipoprotein particle binding	Molecular function	3	0.006313499	0.006313499

**TABLE 8:** GO analysis of DE mRNA in mDCs.

Term	Domain	Count	*P*-value	Corrected *P*-value
Interferon-gamma-mediated signaling pathway	Biological process	5	0.000105145	0.000105145
Regulation of membrane repolarization	Biological process	3	0.000897665	0.000897665
SA node cell to atrial cardiac muscle cell communication	Biological process	2	0.001143106	0.001143106
Cellular response to interferon-gamma	Biological process	5	0.001152483	0.001152483
Atrial cardiac muscle cell membrane repolarization	Biological process	2	0.001392464	0.001392464
Cerebral cortex cell migration	Biological process	3	0.001490903	0.001490903
Telencephalon development	Biological process	6	0.001681684	0.001681684
Response to interferon-gamma	Biological process	5	0.001981297	0.001981297
Membrane repolarization	Biological process	3	0.002157971	0.002157971
Regulation of macrophage cytokine production	Biological process	2	0.002280906	0.002280906
MHC protein complex	Cellular component	3	0.000314384	0.000314384
Integral component of lumenal side of endoplasmic reticulum membrane	Cellular component	3	0.000491764	0.000491764
Lumenal side of endoplasmic reticulum membrane	Cellular component	3	0.000491764	0.000491764
Side of membrane	Cellular component	8	0.000689806	0.000689806
Clathrin-coated endocytic vesicle membrane	Cellular component	3	0.001274169	0.001274169
Plasma membrane protein complex	Cellular component	8	0.002912282	0.002912282
ER to Golgi transport vesicle membrane	Cellular component	3	0.003192827	0.003192827
MHC class II receptor activity	Cellular component	2	0.003242854	0.003242854
Alpha-actinin binding	Cellular component	3	0.003895823	0.003895823
Actinin binding	Cellular component	3	0.006041689	0.006041689
Protein phosphatase 2A binding	Molecular function	3	0.000314384	0.000314384
Lipopolysaccharide binding	Molecular function	3	0.000491764	0.000491764
Peptide antigen binding	Molecular function	3	0.000491764	0.000491764
Oxidoreductase activity, acting on the aldehyde or oxo group of donors, NAD or NADP as acceptor	Molecular function	8	0.000689806	0.000689806
Oxidoreductase activity, acting on the aldehyde or oxo group of donors	Molecular function	3	0.001274169	0.001274169
Protease binding	Molecular function	8	0.002912282	0.002912282
Growth factor receptor binding	Molecular function	3	0.003192827	0.003192827
MHC class II receptor activity	Molecular function	2	0.003242854	0.003242854
Alpha-actinin binding	Molecular function	3	0.003895823	0.003895823
Actinin binding	Molecular function	3	0.006041689	0.006041689

The imDCs migrate to the lymphoid organs that will be matured in the future. They present captured Ag to the naïve T cells (Banchereau and Steinman, 1998). Hence, the imDCs and mDCs had different functions in the presented Ag. In our study, we focused on the immature and mature stages of DCs. In the mDC’ mRNA analysis, 168 mRNAs were identified. Among these DE genes, HLA-B, HLA-DQB1, HLA-DQB2, PTAFR, and F11R were the most significantly upregulated genes in mDCs. HLA-B is the major histocompatibility complex (class I) antigens that present the processed antigens. HLA-DQB1 is the major histocompatibility complex (class II) antigens that have been identified as useful biomarkers of candidacy for effective allergy immunotherapy in patients with AR (Yanming et al., 2019). This result indicates that these cytokines affect the antigen-presenting process in the mDCs. We can determine from the KEGG analysis that there are 12 signaling pathways related to DE mRNAs. The cell adhesion molecules were the useful pathways that contribute to the antigen-presenting function in mDCs (Figures 4E,F), which is consistent with a previous study (Roche and Furuta, 2015). Although many genes have been identified to play an important role in mDCs, but the mechanisms by which HLA-DQB2, PTAFR, and F11R affect mDC in AR are unknown (Tables 9, 10).

**TABLE 9:** lncRNAs in imDCs with the largest fold change.

lncRNA_Accession	FC (abs)	Regulation	Chromosome	Strand	Start	End	Class	Size
TCONS_l2_00030438	6.1069527	Down	chrX	−	5571461	5644346	lncRNA	72885
NON-HSAT016934	5.6181483	Down	chr10	−	127823936	127843874	lncRNA	19938
TCONS_l2_00001274	5.147873	Up	chr1	−	65450880	65451399	lncRNA	519
NON-HSAT016933	4.9528494	Down	chr10	−	127779304	127798357	lncRNA	19053
NON-HSAT059748	4.615665	Down	chr18	+	66817065	66832387	lncRNA	15322
NON-HSAG053933	4.34199	Up	chrX	−	2484070	2527190	lncRNA	43120
NON-HSAG029733	4.2480536	Down	chr2	−	167054881	167055243	lncRNA	362
NON-HSAT093933	4.0003886	Down	chr3	+	188985384	189038493	lncRNA	53109
NON-HSAG036957	3.8607297	Down	chr3	+	188985385	189038493	lncRNA	53108
NON-HSAG055855	3.8563106	Down	chrY	−	21034387	21239448	lncRNA	205061

**TABLE 10:** The characteristics of lncRNAs with the largest fold change in mDCs.

lncRNA_Accession	FC (abs)	Regulation	Chromosome	Strand	Start	End	Class	Size
NON-HSAT016933	4.767738	Down	chr10	−	127779304	127798357	lincRNA	19053
NON-HSAT005246	3.4715514	Down	chr1	−	113068497	113084597	lincRNA	16100
NR_038346.1	3.311445	Down	chr7	+	79082272	79100524	non-coding RNA	18252
NON-HSAG038966	3.027513	Down	chr4	−	141364352	141419531	lincRNA	55179
NON-HSAG037252	3.011491	Up	chr4	−	1165171	1202750	lincRNA	37579
NON-HSAG008700	2.9691923	Up	chr11	−	65556522	65562174	lincRNA	5652
ENST00000450847	2.9003682	Down	chr1	−	248647546	248648785	antisense	1239
ENST00000541885	2.8448925	Down	chr12	−	64900946	64927418	lincRNA	26472
TCONS_00007688	2.8411803	Up	chr4	+	185427281	185436808	lincRNA	9527

Long non-coding RNA is an important component in the mRNA expression profiles (Kopp and Mendell, 2018). Several studies have shown that the differentiation of DCs is closely related to lncRNAs (Pin et al., 2014; Majid et al., 2020). It is confirmed from our studies that lncRNA may play a key role in the development of DC. Our data showed that 172 lncRNAs of imDCs and 104 lncRNAs of mDCs were significantly expressed in patients with AR as compared to that in NP by at least twofold changes. This result helps in studying the AR-related global transcriptome.

Long non-coding RNAs do not have a protein coding function, but they can be used as a new modulator, such as cis- or trans-gene regulating expression, demethylation-promoting effect, and mRNA-processing control were the major mechanisms (Sone et al., 2007; Wahlestedt, 2013; Qiao et al., 2016). To analyze the functions of lncRNAs, mRNA–lncRNA was used to create a co-expression profile to predict the potential functions of the DE lncRNAs of patients with AR. We found that the DE lncRNAs were all included in the results of mRNA–lncRNA chip results.

We used RT-qPCR to validate the mRNA–lncRNA chip results with only one randomly selected transcript, and found that the RT-qPCR results are consistent with the chip results in better understanding the role of lncRNA in the pathogenesis of DC-mediated AR.

In summary, our study proved that several mRNAs and lncRNAs of DC affect a certain process of AR pathogenesis by regulating the target genes. This result points out the direction for future studies on determining and explaining the functions and mechanisms of AR-related mRNA and lncRNA, and provides new therapeutic targets for patients with AR.

## Data Availability Statement

The original contributions presented in the study are included in the article/Supplementary Material. Further inquiries can be directed to the corresponding author/s.

## Ethics Statement

The studies involving human participants were reviewed and approved by the medical and experimental animal ethics committee of Beijing University of Traditional Chinese Medicine. The patients/participants provided their written informed consent to participate in this study.

## Author Contributions

YZ, XC, and YZ carried out the experiments. JW, QW, YZ, XC, and YZ designed the study and edited the manuscript. All authors read and approved the final manuscript.

## Conflict of Interest

The authors declare that the research was conducted in the absence of any commercial or financial relationships that could be construed as a potential conflict of interest.
