# Supplementary material for: Long Non-coding RNAs and mRNAs Expression Profiles of Monocyte-Derived Dendritic Cells From PBMCs in AR
Source: Front Cell Dev Biol. 2021 Feb 9;9:636477. doi: 10.3389/fcell.2021.636477 (PMC7906227; doi:10.3389/fcell.2021.636477)
Supplement: Supplementary file 1 [file Table_1.DOCX]

**Table S1 Primers used for qRT-PCR**

| **Gene** | **Forward primer (5' to 3')** | **Reverse primer (5' to 3')** | **Product(bp)** |  |
| --- | --- | --- | --- | --- |
| ITGAV | TGCCTGTGTGGGTGATCATT | TTCTTCTTGAGGTGGCCGGA | 116 |  |
| MARCO | AAGGGCCCTGTACAAAGTGG | TGAAAGGGTTTCCGGGTCAG | 176 |  |
| CD36 | CGGCTGCAGGTCAACCTATT | TTCTATCAGGCCAAGGAGGT | 180 |  |
| IFNB1 | TTTGCTCTGGCACAACAGGT | AAGCCTCCCATTCAATTGCC | 195 |  |
| KIR2DS3 | CCCCAGACACCTACACGTTC | TGACACCTCCTGATGGTCCT | 187 |  |
| PTAFR | CATTTCCTCCCAGGGGTGAC | TCATGTGGCTCCATTGCTGT | 197 |  |
| F11R | TGGTTTGCCTATAGCCGAGG | CAGATGATAGGCGGTGAGCC | 157 |  |
| HLA-B | CTGTTCCCATGCTGACCTGT | GCAGCTCAGTGCACGTAAAG | 106 |  |
| HLA-C | GGTGGAAAAGGAGGGAGCTG | ACACATTCAGGTGCCTTTGC | 190 |  |
| HLA-DQB1 | CTCAAGGGCCTCCACCAG | TGCAGGAAGCAGAGTCACAG | 227 |  |
| GAPDH | GGTGAAGGTCGGAGTCAACG | CAAAGTTGTCATGGATGACC | 416 |  |
| \| NONHSAT059748 \| \| --- \| | ATCCCCTTGGTGCTGTCTCAT | CGGTGCTACTCTTCTTCCAGG | 114 |  |
| NONHSAG046717 | AAAGGATGAATCAAACCCACAGGC | ACGAACCAAGCCTCACGACA | 226 |  |
| NONHSAT089067 | CATACTCAACAGCCTTCCAGTCC | ACTTACTCCTGCCTCTATGGTGA | 206 |  |
| NONHSAT024276 | TCCCTCCCTTCCTCCCCATA | ACTCCGTTTCATCATTAACCGACC | 224 |  |
| NONHSAT098958 | AGGAGGTGATGCTGGAACAGG | GACTCGGGAACTTAATTGGGAC | 117 |  |
